# A Novel and Feasible Intracorporeal Esophagojejunostomy Anastomosis in Totally Laparoscopic Total Gastrectomy Surgery: Sutureless L-Shape with Endoscopic Assistance (SLEJ)

**DOI:** 10.3390/medicina61050795

**Published:** 2025-04-25

**Authors:** Ibrahim Burak Bahcecioglu, Sumeyra Guler, Sevket Baris Morkavuk, Mujdat Turan, Gokhan Giray Akgul, Mirac Baris Erzincan, Kubilay Kenan Ozluk, Osman Bardakci, Mehmet Ali Gulcelik

**Affiliations:** 1 Department of Surgical Oncology, Gulhane Research and Training Hospital, Ankara 06010, Turkey; dr.ibb@hotmail.com (I.B.B.); guler.sumeyra@yahoo.com (S.G.); bariserzincan@hotmail.com (M.B.E.); drkenanozluk@gmail.com (K.K.O.); drosmanbardakci@gmail.com (O.B.); 2 Department of Surgical Oncology, Gulhane Faculty of Medicine, Health Sciences University, Ankara Gulhane Research and Training Hospital, Ankara 06010, Turkey; girayakgul@hotmail.com (G.G.A.); mehmetali.gulcelik@sbu.edu.tr (M.A.G.); 3 Department of General Surgery, Gulhane Faculty of Medicine, Health Sciences University, Ankara Gulhane Research and Training Hospital, Ankara 06010, Turkey; mujdatturan84@hotmail.com

**Keywords:** endoscopic assistance, intracorporeal, laparoscopic esophagojejunostomy, sutureless

## Abstract

*Background and Objectives*: In contrast to the standardization of laparoscopic gastrectomy techniques, the complexity of intracorporeal anastomosis techniques in totally laparoscopic total gastrectomy, the lack of standardization, the positional challenges posed by working in a confined space, and varying complication rates have prevented a consensus on the optimal intracorporeal digestive tract reconstruction method. Selecting an appropriate reconstruction method for esophagojejunostomy is crucial for a successful surgical outcome. This study aims to define a modified anastomotic technique for TLTG and share our experience with this technique. *Materials and Methods*: A total of 21 patients who underwent TLTG with D2 LND between July 2024 and December 2024 using the sutureless L-shape esophagojejunostomy (SLEJ) technique at the Surgical Oncology Clinic of Gulhane Training and Research Hospital due to gastric cancer were included in the study. In our technique, gastrectomy, lymph node dissection, anastomosis preparation, esophagojejunostomy anastomosis, and enteroenterostomy anastomosis were all performed laparoscopically and intracorporeally. *Results*: The mean operative time was 180.48 min, with a mean EJ anastomosis duration of 40.24 min. In the standard technique, two Endo GIA™ staplers were used for pyloric and small bowel transection, two for EJ anastomosis, and one for intracorporeal jejunojejunostomy. In only one patient, three staplers were used for anastomosis. Therefore, the average number of staplers was 5.05, with a mean of 2.05 staplers used for anastomosis. The mean hospital stay was 8.19 days, and there were no mortalities. The number of patients with an anastomotic leakage was 1. Since the patient’s general condition remained stable, percutaneous drainage or laparotomy was not planned. The patients’ esophagojejunostomy anastomotic leak was classified as Class 1 and Grade 3a according to the Clavien–Dindo classification. The average size of our widest incision was 3.28 cm, and surgical site infections were developed in two patients. *Conclusions*: Sutureless L-Shape With Endoscopic Assistance (SLEJ) is an easily applicable, technically simpler, shorter-in-duration, easier-to-learn, and safer intracorporeal EJ anastomosis technique with a low rate of postoperative complications.

## 1. Introduction

Gastric cancer is a common malignant tumor that ranks fifth worldwide in both incidence and mortality [1]. Over the past 30 years, the successful eradication of helicobacter pylori infection, which has been primarily been held responsible for distal gastric cancer etiology, along with a concurrent increase in autoimmune gastritis and dysbiosis of the gastric microbiome factors implicated in proximal gastric cancer have led to a significant rise in the incidence of proximal gastric cancer [2,3,4,5,6]. Despite the increasing trend toward a multidisciplinary approach, surgery remains the primary treatment for proximal gastric adenocarcinoma. Total gastrectomy is a preferred surgical technique for treating conditions such as proximally located gastric cancer and large gastric ulcers. In recent years, the increasing popularity of minimally invasive surgery, advancements in laparoscopic instruments and techniques, and growing surgical experience have enabled total laparoscopic total gastrectomy (TLTG) to be performed with increasing frequency. Compared to open surgery, the laparoscopic approach is less invasive, offering advantages such as shorter hospital stays, reduced bleeding, faster recovery times, and favorable cosmetic outcomes [7]. However, due to technical challenges associated with the esophagojejunostomy (EJ) anastomosis procedure and concerns regarding inherent oncological safety, TLTG has not yet been as widely accepted as laparoscopic-assisted total gastrectomy (LATG). One of the most technically demanding aspects of this surgical procedure is the esophagojejunostomy (EJ), which establishes the connection between the esophagus and jejunum. If not performed properly, EJ poses a risk for postoperative complications such as anastomotic leakage, stenosis, rotation, and stricture. The incidence of anastomotic complications such as postoperative leakage and stenosis is relatively higher in laparoscopic procedures compared to open techniques [8].

The resection of the primary lesion and associated lymph nodes remains a major oncological safety concern in laparoscopic and robotic surgery, forming the primary argument for surgeons advocating open surgery. However, increasing experience with laparoscopic and robotic techniques and the widespread adoption of minimally invasive methods have largely addressed these concerns. Zheng CY et al. proposed the “enjoyable space” technique for lymph node dissection (LND) and complete mesogastric excision (CME), which has been modified by our team and found to be a valuable guide for achieving accurate and complete resection [9].

Anastomotic complications not only reduce survival rates but also significantly affect long-term quality of life [10]. Therefore, selecting an appropriate reconstruction method for EJ is crucial for a successful surgical outcome. Intracorporeal digestive tract reconstruction is a challenging step in TLTG, and due to the technical difficulty of intracorporeal anastomosis, many surgeons prefer LATG, where extracorporeal anastomosis is performed through a mini-laparotomy.

In LATG, the end-to-end Roux-en-Y EJ using a circular stapler is widely accepted as the standard reconstruction method due to its low risk of leakage. However, this technique has major challenges, including the difficulty of placing a purse-string suture around the distal esophageal stump in deep localization after resection and inserting and securing the anvil head through a narrow space [11,12]. These challenges have led to the development of techniques using a reverse puncture device or an OrVil system. Still, a mini-laparotomy remains necessary for proper placement of the circular shaft in LATG [13,14].

The complexity of intracorporeal anastomosis techniques in TLTG, the lack of standardization, the positional challenges posed by working in a confined space, and varying complication rates have prevented a consensus on the optimal intracorporeal digestive tract reconstruction method [15,16,17,18,19,20]. Among the most commonly used anastomotic techniques are overlap anastomosis, OrVil™ anastomosis, π-shaped anastomosis, and their modified variations. However, there are limited studies comparing these anastomosis techniques with one another and evaluating the advantages of TLTG over LATG.

This study aims to define a modified anastomotic technique for TLTG, share our experience with this technique, compare it with established methods, and assess its safety and feasibility relative to LATG.

## 2. Patients and Methods

### 2.1. Patients

All patients who underwent TLTG with D2 LND between July 2024 and December 2024 using the sutureless L-shape esophagojejunostomy (SLEJ) technique at the Surgical Oncology Clinic of Gulhane Training and Research Hospital due to gastric cancer were included in the study. All patients underwent upper gastrointestinal endoscopy and histopathological examination for diagnostic purposes, along with staging via oral and intravenous contrast-enhanced thoracoabdominal pelvic CT, in accordance with our standard clinical protocol. For patients with T2 or higher tumors and/or nodal disease, surgery was performed following neoadjuvant chemotherapy [21]. All patients were informed about the procedure and provided written informed consent. This study was approved by the Scientific Research and Evaluation Board of the Gulhane Training and Research Hospital (Approval No.: 50687469-771-263793437).

Patients who did not undergo TLTG, those who underwent digestive tract reconstruction using techniques other than SLEJ anastomosis, and those with a postoperative clinical follow-up of less than three months were excluded from the study. The data collected from patients included general characteristics (age, sex, body mass index, presence of comorbidities, smoking status, weight loss, presence/absence of neoadjuvant therapy), surgical outcomes (operation duration, anastomosis duration, intraoperative blood loss during anastomosis, number of staplers used, cost analysis, hospitalization duration, pathological evaluation, presence of complications, and Clavien–Dindo classification), and postoperative outcomes (early postoperative (three-month) clinical assessment).

### 2.2. General Procedure

All operations were performed by the same team. This prevented heterogeneity in terms of technique and time. The operation was performed by a professor, an associate professor, a specialist, and a fellow. The endoscopy was performed by two associate professors. All the doctors who performed the surgery and endoscopy are specialists in general surgery and surgical oncology. The surgery is initiated in the supine position with both legs open under intratracheal general anesthesia. The operator is positioned on the left side of the patient, the assistant on the right, and the camera operator between the legs. The first 10 mm trocar is inserted into the abdomen using an open technique via a subumbilical midline incision, and CO_2_ insufflation is initiated. Pneumoperitoneum is established, with the intra-abdominal pressure set to 10–12 mmHg. The patient is then placed in a 30–40° reverse Trendelenburg position. Two 5 mm assistant ports are placed on the right side, with the superior boundary being 2 cm inferior to the costal margin. On the left side, one 5 mm trocar is positioned superolaterally at a minimum distance of 2 cm from the costal margin, and one 10 mm trocar is placed inferomedially.

### 2.3. D2 Lymph Node Dissection (D2 LND)

After intra-abdominal exploration, omentectomy dissection and gastric skeletonization are deferred. The gastrocolic ligament (GCL) is opened using a bipolar electrothermal coagulation device, namely, the LigaSure™ Sealer/Divider, at the lateral side of the Barkow arch, starting from the distal one-third of the greater curvature. The left gastroepiploic artery (LGEA), posterior gastric artery (PGA), and arteria gastrica breves (AGB) are ligated along the greater curvature. The stomach is freed from the omentum by separating the gastrosplenic ligament (GSL) and gastro-phrenic ligament (GPL) up to the left diaphragmatic crus, including the fundus (LN stations 2, 4sa, and 4sb).

Preoperatively, all radiological imaging is routinely evaluated in our clinic, both by our surgical team and a single experienced radiologist to anticipate possible vascular variations. A review by Cirocchi et al., analyzing 57 studies and 19,284 patients, identified the prevalence of an accessory (5.5%) and aberrant (8.2%) left hepatic artery (LHA) originating from the left gastric artery (LGA) as 13% [22]. The preservation of aberrant and accessory LHA does not compromise oncologic safety and can be applied both laparoscopically and robotically [23,24]. While some researchers argue that the ligation of the accessory LHA is harmless and the aberrant LHA should be preserved, our clinical approach prioritizes awareness of vascular variations before surgery and preserving both accessory and aberrant LHA unless absolutely necessary [25,26,27,28].

The GCL extending to the distal one-third of the greater curvature is also dissected anteriorly to the transverse mesocolon and mobilized down to the root of the right gastroepiploic artery (RGEA) and right gastroepiploic vein (RGEV) (LN station 4sd). After clipping and ligating the RGEA and RGEV, gastric mobilization continues to the pylorus (LN station 6). To explore both D2 LND and potential vascular variations, dissection extends from the anterior pancreas fascia to the dorsal mesogastrium. The gastroduodenal artery (GDA) is identified and traced to the common hepatic artery (CHA), followed by exposure of the right gastric artery (RGA). Before ligating the RGA from an inferior approach, the gastrohepatic ligament (GHL) is opened, and the lesser curvature is mobilized from the pylorus inferiorly to the right diaphragmatic crus superiorly (LN stations 1, 3, and 5). The lesser omentum is dissected along the inferior liver border, and the lymph nodes anterior to the hepatoduodenal ligament along the arteria hepatica propria (HPA) and portal vein (PV) are dissected en bloc (LN station 12a). The suspension of the posterior gastric wall facilitates perigastric fascia access, allowing dissection along the surfaces of the GDA and CHA (LN station 8a). The RGA, which can also be accessed inferiorly via the suspension of the gastric wall, is clipped and divided from the suprapyloric region via an inferior approach (LN station 5).

After completing the dissection of the inferior border, the 10 mm medioinferior trocar on the left side is replaced with a 12 mm trocar to allow for stronger gastric traction over the celiac trunk (CT). Since we believe this facilitates the procedure, the postpyloric duodenum is transected using an Endo GIA™ stapler without delaying it until the end of the D2 lymph node dissection (LND).

The fascia surrounding the RGA and GDA is dissected, and lymphatic tissues along the mid-distal pancreas up to the celiac trunk are resected. The left gastric vein (LGV) is first isolated, followed by the LGA, which is freed up to its root at the CT and then clipped and ligated (LN stations 7 and 9). After LGA and CT dissection, the splenic artery (SA) is traced along its proximal course, with surrounding adipose and connective tissues, including lymphatic structures, resected en bloc with the specimen (LN stations 11p and 11d). Finally, at the gastroesophageal junction (GEJ), the esophagophrenic ligament and both vagus nerves are transected, ensuring complete mobilization of the distal esophagus. The GEJ, which remains the sole continuity of the stomach, is mobilized through blunt dissection with gastric traction, achieving at least 8–10 cm of mobilization at the lower esophagus from the cardia.

### 2.4. Omentectomy

Although omentectomy has not been proven to provide a survival advantage in gastric cancer patients, it remains a standard treatment for advanced gastric cancer [29]. We believe that postponing total omentectomy until after gastric skeletonization and D2 LND minimizes omental hemorrhage, facilitating both exploration and traction. Additionally, our experience suggests that the liberated tissues, particularly the stomach, cause volume-related restrictions in the subdiaphragmatic space, especially during anastomosis. Therefore, we advocate for performing omentectomy after gastric resection. Once the stomach is entirely mobilized up to the GEJ, omentectomy is initiated at the midpoint of the transverse colon and continues laterally to the hepatic flexure on the right and the splenic flexure on the left, completing the detachment from the transverse colon.

### 2.5. Preparation for Anastomosis

The jejunal segment for esophagojejunostomy (EJ) is typically identified 30–60 cm distal to the ligament of Treitz, though this distance may vary depending on individual anatomical variations. Generally, the segment falls within the 30–40 cm range; however, the primary determining criterion is selecting the jejunal segment that reaches the gastroesophageal junction (GEJ) with the least tension. After isolating the mesentery of the identified segment using LigaSure™, the jejunum is divided with Endo GIA™. The distal jejunal limb is positioned in the right quadrant of the abdomen, while the proximal limb is placed in the left quadrant. An enterotomy is performed using a hook approximately 5–7 cm distal to the transection line on the distal limb, parallel to the antimesenteric axis of the jejunum. The segment is then gently transferred into the anastomosis site in an antecolic manner using two graspers, both from the transection line and the mesentery.

In cases where the esophagus is transected, traction during intracorporeal anastomosis is achieved using suspension sutures, laparoscopic Babcock forceps, or graspers. However, all these instruments directly contact the esophageal segment undergoing anastomosis, posing a risk of trauma. Additionally, whether graspers or suspension sutures are used, the manipulation of the transected lumen requires suspension from two separate ends, unnecessarily blocking two trocars. The stomach serves as a natural, atraumatic, and strong instrument for esophageal traction through a single trocar. Furthermore, single-trocar manipulation of the stomach can facilitate esophageal rotation, which is not possible with the conventional side-to-side technique.

### 2.6. Endoscopic Guidance

In the side-to-side linear stapled esophagojejunostomy (SLEJ) anastomosis technique, esophagotomy is performed under endoscopic guidance to prevent full-thickness esophagotomy that could lead to submucosal dissection, minimize contralateral wall injury, and ensure a precise macroscopic resection margin, especially in GEJ tumors [30]. By rotating the stomach 30–60 degrees clockwise, esophagotomy is created on the right posterolateral side of the esophagus, parallel to its axis, using a hook, with endoscopic guidance. The incision is made approximately 8–10 mm in length—just enough to accommodate the thick jaw of the Endo GIA™ stapler. In our anastomosis technique, which we consider to be highly dependent on endoscopic guidance, we recommend using endoscopy both as a guide during EJ construction and stapler firing for defect closure in esophagotomy, jejunotomy, and pyloric transection. Additionally, post-anastomosis endoscopy is advised for control purposes.

### 2.7. Anastomosis Technique

**Figure 1:**
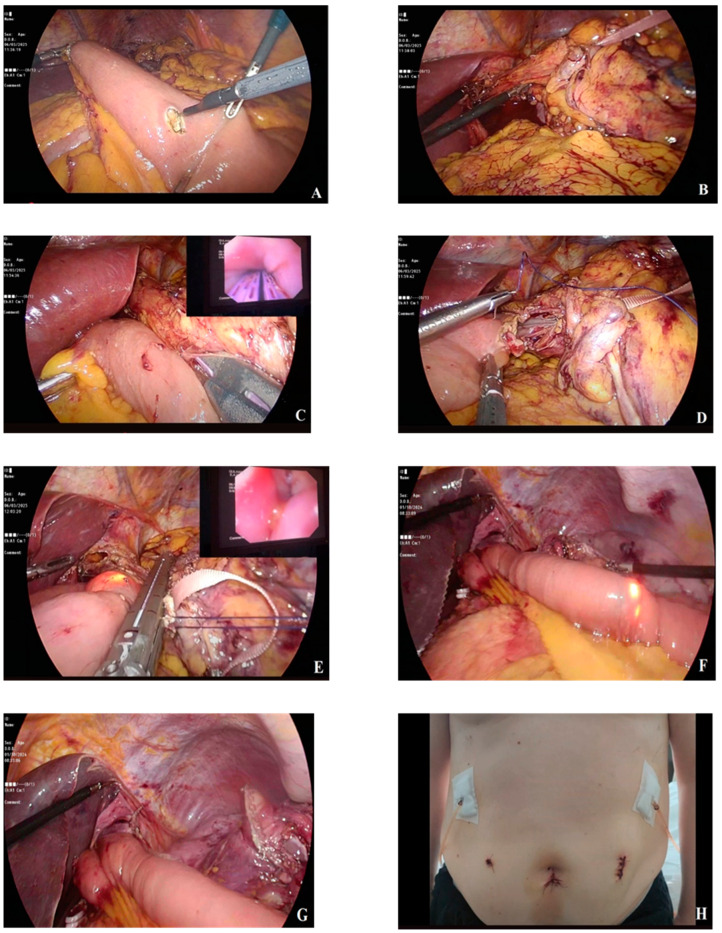
Pictures of the anastomosis technique steps. (**A**) Performing enterotomy, (**B**) gastric traction for preparation of anastomosis, (**C**) performing side-to-side EJ anastomosis with endoscopic guidance, (**D**) the placement of a Vicryl suture for the suspension of the jejunostomy defect, (**E**) the closure of both the esophagotomy and jejunotomy defects with endoscopic guidance, (**F**) the control of anastomosis width by endoscopy, (**G**) the checking of esophageal and intestinal rotation, and (**H**) the suturation of trocar incisions.

**Figure 2:**
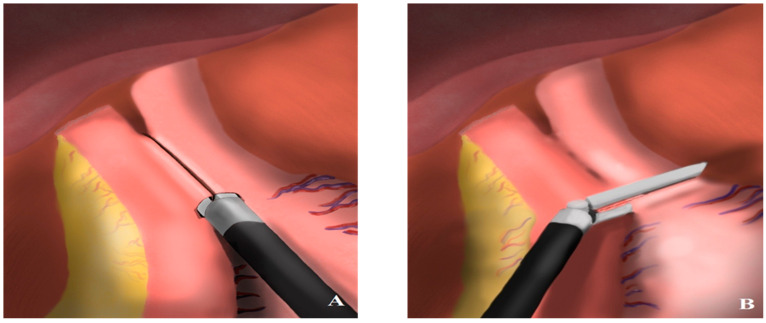
Illustrative technical draw. (**A**) Side-to-side EJ anastomosis; (**B**) closure of the esophagotomy and jejunotomy defects.

According to Inaba et al., the overlap method, a linear stapled reconstruction technique, provides an improved view, ensures an isoperistaltic jejunal orientation, and allows for a higher anastomosis position, making it a more favorable approach for esophagojejunostomy [14]. In conventional side-to-side anastomosis, the distal esophageal stump is closed with a second Endo GIA™ stapler after EJ firing, while the jejunotomy defect is closed with manual suturing [31]. This intracorporeal suturing significantly prolongs the anastomosis duration and introduces risks, such as passing sutures through the posterior wall of the jejunum, causing jejunal rotation, and increasing the likelihood of jejunal stricture. In our described technique, following the firing of the EJ stapler, a second stapler is used in a single step to perform pyloric transection while simultaneously closing the esophagotomy and jejunotomy defects. This allows the entire anastomosis to be completed using only two staplers. The proper positioning of the jejunotomy defect, just outside the stapler’s firing line, is ensured using a jejunal suspension suture. Once the endoscope is advanced into the jejunum, a single 2/0 Vicryl suture is placed 1–2 mm medial to the jejunotomy defect for suspension, without requiring a knot. While the distal jejunal limb is retracted mediocaudally, the stomach is pulled cranially toward the left, and the suspension suture is pulled laterally. This coordinated traction enables a single stapler to simultaneously transect the distal esophagus and close both the esophagotomy and jejunotomy defects. Endoscopic guidance in anastomosis not only prevents technical complications such as rotation and anastomotic stricture but also eliminates issues such as submucosal anastomosis and intraluminal anastomotic bleeding through intraluminal evaluation. Another advantage of this technique is the absence of any defect requiring suturing, which significantly shortens both the anastomosis and overall operation time. In some side-to-side EJ anastomosis techniques, the jejunotomy defect is positioned caudally, resulting in an anisoperistaltic configuration, where passage continuity relies on a modified π-loop deformity [12,32]. This configuration delays drainage until the EJ pouch is entirely filled. In contrast, our SLEJ anastomosis technique, in which the jejunotomy defect is placed cranially, maintains an isoperistaltic configuration, eliminating any jejunal pouch formation. As a result, the reconstructed digestive tract mimics normal anatomical function more closely. In this tension-free, endoscopically guided anastomosis, additional serosal suturing for reinforcement was not required. Particularly in cases with distal esophageal invasion, we strongly recommend intraoperative endoscopic assistance for macroscopic determination of the proximal surgical resection margin in our intracorporeal anastomosis technique (Figure 1 and Figure 2).

### 2.8. Biliopancreatic Limb

Approximately 30–40 cm distal to the EJ anastomosis, a jejunojejunostomy is performed in an anisoperistaltic side-to-side fashion using the Endo GIA™ stapler (Covidien, Mansfield, MA, USA), following the creation of jejunotomies on both limbs at the left lateral aspect. The approximately 2 cm jejunotomy defect is then closed laparoscopically using a needle holder.

### 2.9. Specimen Retrieval

The excised omentum, stomach, and D2 lymphadenectomy (LND) specimens are extracted via the 12 mm trocar site located inferomedially on the left abdomen using an O-wound protector/retractor. The necessity to enlarge the trocar site is typically correlated with the patient’s body mass index and tumor size, generally requiring an extension of approximately 3 cm. After specimen retrieval, pneumoperitoneum is re-established, and hemostasis is confirmed. Two Jackson-Pratt drains are placed: one extending to the duodenal stump through the right inferior 5 mm trocar site and the other extending to the EJ anastomosis line via the left superior 5 mm trocar site. Following drain placement, the port sites are sequentially closed.

### 2.10. Postoperative Care and Follow-Up

Venous thromboembolism (VTE), which includes deep vein thrombosis (DVT) and pulmonary thromboembolism (PTE), is one of the preventable major causes of postoperative morbidity and mortality [33]. We evaluate the risk of thrombosis in all patients using the Caprini scoring system. Even before considering individual patient characteristics, the presence of gastric cancer and a planned laparoscopic surgery lasting more than 45 min automatically categorizes patients as at least moderate-risk with a score of ≥4 [34]. Due to this risk, all patients receive early ambulation, mechanical methods, and pharmacoprophylaxis with low-molecular-weight heparin.

Postoperative patient monitoring is conducted in the clinic or intensive care unit, depending on the preoperative ASA score (The American Society of Anesthesiologists Physical Status Score) and perioperative patient monitoring data, as assessed by the experienced anesthesiologist involved in intraoperative monitoring [35]. Patients requiring intensive care unit (ICU) follow-up are transferred to the ward the next day unless a pathological condition arises. In clinical follow-up, patients are started on oral intake with water on the standard postoperative day 4. No methylene blue or other leakage tests are required before oral intake.

A single prophylactic dose of 1 g cefazolin is administered before surgery, and for patients with a β-lactam allergy, 600 mg clindamycin/quinolone is used. No postoperative continuation of these antibiotics is necessary.

Regarding drain management, the right-side drain, which monitors the duodenal stump, is removed on postoperative days 4–6 if the drainage volume is <50 cc/24 h and its characteristics are normal. The left-side drain, which monitors the EJ anastomosis, is removed on postoperative days 5–7, provided there is no change in color or odor and the drainage remains serous or serohemorrhagic.

### 2.11. Video Presentation

**Figure 3:**
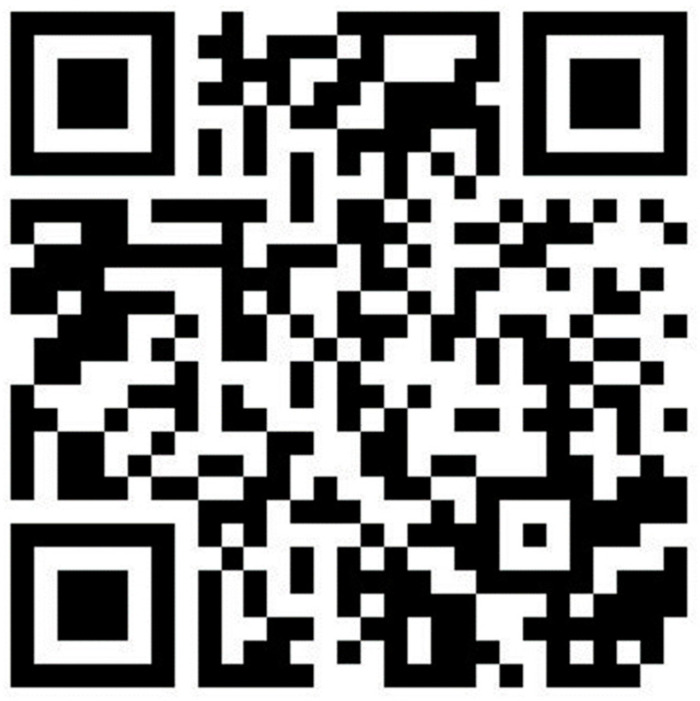
QR code of video demonstration. https://youtu.be/bLGxSlRSP9Q?si=T500aEF_eZAb-tQ4 (accessed 1 March 2025).

To enhance the comprehensibility of our described novel anastomotic technique, SLEJ, we integrate a supplementary video demonstration into this manuscript. This video, recorded during the procedure, provides a detailed visual representation of the surgical steps, facilitating a clearer understanding of the technique. A QR code linking to the video has been generated and is included in the manuscript (Figure 3). This approach aims to complement the textual and illustrative content, offering readers an interactive and practical perspective on the described method.

### 2.12. Statistical Analysis

Statistical analysis was performed using SPSS v22.0 software (SPSS Inc., Chicago, IL, USA). Normality tests for continuous variables were conducted using the Kolmogorov–Smirnov and Shapiro–Wilk tests. Continuous variables are presented as mean ± standard deviation (SD) or median (range), while categorical variables are expressed as numbers (n) and percentages (%).

## 3. Results

### 3.1. Patient Characteristics

A total of 24 patients underwent surgery using our technique. Since we planned a minimum follow-up period of 3 months, 21 patients were included in our case series. Of these patients, 13 were male and 8 were female. The average age was 59.61 years for men and 58 years for women. The mean height was 169.24 cm, and the mean body weight was 75.39 kg. In their medical history, 61.9% (13 patients) were smokers. Five patients had cardiovascular disease, three had diabetes mellitus, and two had a history of previous cancer surgery. Preoperative evaluations showed no signs of anemia or cachexia in any patient. The mean hemoglobin level was 11.78 g/dL, and the mean albumin level was 4.19 g/dL. Among the patients, 15 were classified as locally advanced cases and underwent surgery after completing neoadjuvant therapy (Table 1).

### 3.2. Tumor and Pathology Results

None of the patients had a Siewert type 1 tumor localization. Of the patients, 9 had Siewert type 2 tumors, while 12 had Siewert type 3 tumors. Regarding histological classification, 12 patients had classic variant adenocarcinoma, 5 had signet-ring cell adenocarcinoma, and 4 had neuroendocrine tumors (MiNENs). Tumor differentiation was grade 1 in seven patients and grade 3 in eight patients. All patients diagnosed with neuroendocrine tumors were classified as grade 3. The mean number of dissected lymph nodes was 24.81, and the mean number of metastatic lymph nodes was 3.14. Tumor staging revealed that 6 patients had stage T4a, 3 had stage T3, and 12 had early-stage T tumors. Postoperative pathological evaluation identified nine patients with N0 and four with N3 status (Table 1).

### 3.3. Surgical Technique Early Postoperative Outcomes

The esophagojejunostomies of all included patients were performed using our described SLEJ technique. None of the patients required conversion to open surgery. The mean operative time was 180.48 min, with a mean EJ anastomosis duration of 40.24 min. Initially, the anastomosis time ranged between 45 and 50 min, but with increasing experience, it was reduced to 30 min. As the anastomosis time decreased, the overall surgery duration was reduced from 200–210 min to 160–170 min. In our standard technique, two Endo GIA™ staplers were used for pyloric and small bowel transection, two for EJ anastomosis, and one for intracorporeal jejunojejunostomy. In only one patient, three staplers were used for anastomosis, increasing the total stapler count to 5.05 on average, with a mean of 2.05 staplers used for anastomosis. A cost analysis based on stapler usage revealed that the cost of the SLEJ anastomosis technique was USD 155. Due to the use of three staplers in one patient, the average cost increased to USD 158.57. The mean intraoperative blood loss was 45.71 mL, and no blood transfusions were required. There were no median or other laparotomy incisions. The transected stomach specimen was extracted through an extended 12 mm trocar incision in the left abdomen using an O-wound protector/retractor. The incision size varied between 3 and 4.5 cm depending on the patient’s body mass index, with an average incision size of 3.28 cm. The mean hospital stay was 8.19 days. There were no mortalities, and the average weight loss in the first three months postoperatively was 3.24 kg (Table 2).

### 3.4. Postoperative General and Anastomotic Technique Complications

Postoperative early complications developed in 10 (47.6%) of our 21 patients (Table 3). Nine of these complications were general, while one was related to the EJ anastomosis. Among the general complications, pulmonary system complications were the most common. Four patients developed right lower lobe atelectasis, two had bilateral lower lobe atelectasis, and one patient had right pleural effusion. Two patients with atelectasis accompanied by pneumonia underwent deep tracheopulmonary aspiration via bronchoscopy by the thoracic surgery team, while the patient with pleural effusion underwent pleural aspiration. The remaining patients were followed up with conventional methods. Two patients who developed surgical site infections were managed with wound irrigation and antibiotic therapy. In one patient with an anastomotic complication, a change in drain content color and an increase in inflammatory laboratory parameters were observed on postoperative day 3, prompting an oral contrast-enhanced thoracoabdominal CT scan. The CT revealed a minimal extraluminal contrast leak and a free fluid collection around the EJ anastomosis. Since the patient’s general condition remained stable, percutaneous drainage or laparotomy was not planned. The patient’s antibiotic regimen was adjusted, and TPN n7 was initiated. As the inflammatory parameters regressed during follow-up, oral methylene blue was administered on postoperative day 9. Under normal conditions, oral intake is initiated with fluids on postoperative day 4. Once no methylene blue leakage was observed in the drain, liquid nutrition was started. Based on the current treatment and follow-up protocol, the patient’s esophagojejunostomy anastomotic leak was classified as Class 1 and Grade 3a according to the Clavien–Dindo classification [36]. The patient was discharged on postoperative day 14. All patients underwent upper gastrointestinal endoscopy at postoperative month 3 for anastomotic assessment. None of the patients showed signs of granulation tissue or anastomotic stricture related to an anastomotic leak. In all patients undergoing our SLEJ technique, endoscopy confirmed that the anastomotic diameter was sufficient for oral intake.

## 4. Discussion

Cancer was first documented around 3000 BCE in the Edwin Smith Papyrus, while gastric cancer was first mentioned in the Ebers Papyrus, one of Egypt’s most significant medical texts, dating back to approximately 1600 BCE [37]. Despite such an early historical record, modern treatment approaches only began in the late 19th century. The first attempts at gastrectomy for gastric cancer were made by French surgeon Jules Péan in 1879 and Polish surgeon Ludwig Rydygier in 1880, albeit both were unsuccessful. A major milestone in gastric surgery was reached with Billroth’s subtotal gastrectomy in 1881 and Karl Schlatter’s first total gastrectomy in 1897 [38]. For nearly a century, open gastrectomy has been considered the cornerstone of surgical treatment for gastric cancer. However, advancements in minimally invasive surgery have also influenced gastric cancer surgery. Approximately 60 years after Carl Fervers’s 1933 introduction of “laparoscopic adhesiolysis,” the first surgical laparoscopy in a modern sense, Kitano performed the first laparoscopic-assisted gastrectomy in Japan [39,40]. The improved postoperative comfort and favorable oncological outcomes of laparoscopic total gastrectomy (LTG) have facilitated its acceptance among surgeons. Today, lymph node dissection and gastrectomy extent in gastric cancer surgery have been standardized regardless of whether conventional or laparoscopic surgery is performed. However, there is still no consensus regarding the technical aspects of laparoscopic gastrectomy. In particular, the anastomotic stage following gastrectomy remains a topic of ongoing discussion, with various techniques being explored by different surgeons. The esophagojejunostomy (EJ) anastomosis, performed after total gastrectomy, is the most widely discussed technique and is the step where many novel techniques are proposed. Even in conventional total gastrectomy, the EJ anastomosis is a critical phase, significantly impacting early postoperative morbidity. In LTG, performing the EJ anastomosis presents a major technical challenge, even for experienced surgeons. This technical difficulty is a key reason why totally laparoscopic total gastrectomy (TLTG) is not as widely performed as totally laparoscopic distal gastrectomy (TLDG) or laparoscopy-assisted total gastrectomy (LATG). Various techniques have been introduced to overcome these challenges, but none have been definitively proven superior to others [31,41]. The common goals of these techniques are lower complication rates, reduced costs, and a faster return to daily activities. In this study, we aimed to introduce our newly developed totally laparoscopic sutureless L-Shape esophagojejunostomy anastomosis technique to the academic community with visual evidence and to compare its early postoperative outcomes with the existing literature.

The most significant morbidity factor following TLTG is EJ anastomotic leakage, primarily due to the technical difficulty of laparoscopic anastomosis and the esophagus’s fragile histo-anatomical structure. The esophagus is a luminal organ composed of mucosal and muscular layers without a serosal layer. As a result, traction during anastomosis can easily lead to unintended injuries to the esophageal tissue. Furthermore, the esophagus lacks a distinct arterial and venous structure, meaning that collateral circulation can be compromised during such injuries, further impairing anastomotic vascularization. To counteract these challenges, we designed and developed a novel anastomotic technique. In our anastomosis technique, the stomach is not transected from the esophagus after dissection, allowing it to be used for traction during anastomosis. This approach helps prevent unnecessary tissue loss and disruption of vascular circulation in the esophagus. A review of the literature reveals a wide variety of EJ anastomotic techniques. A meta-analysis examining the six most commonly used techniques compared their rates of anastomotic leakage and stricture formation [42]. The highest leakage rate was observed with the OrVil anastomosis technique (3.2%), while the lowest leakage rate was reported in the intracorporeal linear end-to-end anastomosis technique. In our anastomotic technique, leakage occurred in only one patient, which we attributed to the additional use of a stapler and overlapping stapler lines. This patient’s anastomotic leakage was managed with conventional methods, and the patient was discharged without the need for laparotomy or additional surgical intervention. In contrast, none of the patients who underwent our standard SLEJ technique experienced anastomotic leakage.

An essential step in our SLEJ anastomosis technique is the use of an endoscopy assistant. Endoscopy serves multiple beneficial functions during and after the anastomosis phase. First, as in sleeve gastrectomy, it confirms that the EJ anastomosis is of adequate width to allow the passage of a scope. Anastomotic stricture is one of the significant complications in intracorporeal EJ anastomosis, as reported in the literature. The incidence of postoperative EJ anastomotic stricture was reported as 5.9% in the study by Takayama et al. and 9.1% in the study by Wu et al. [12,43]. However, in our technique, no findings suggestive of anastomotic stricture were observed in the early postoperative period or at the three-month follow-up. Another advantage of endoscopic guidance is that it allows direct visualization of the staple line after anastomosis, enabling hemostasis control. Bleeding foci at the anastomotic site can be cauterized with endoscopic assistance or controlled using clips. Possible endoluminal bleeding, hemoglobin drop, deterioration in vital signs, or gastrointestinal bleeding can be detected in the early period before clinical symptoms appear. In only one patient, a bleeding focus was identified intraoperatively at the anastomotic site and was cauterized endoscopically. No cases of postoperative anastomotic bleeding were encountered. A literature review revealed that various studies have reported an anastomotic bleeding rate of 9% [44,45]. Another advantage of the endoscopy assistant is that it ensures a sufficient surgical margin by allowing direct visualization of the tumor tissue. As a result, intraoperative frozen section analysis was not required in any of our patients. Our pathology results confirmed clear surgical margins in all cases. This advantage provides both time and cost savings. Finally, the most significant advantage of endoscopy is its ability to prevent submucosal anastomosis (pseudo-anastomosis), a complication that directly impacts morbidity and mortality [46]. During the anastomosis, the EndoGIA stapler is visible, and the intraluminal anastomotic width and length can be assessed. This helps prevent false lumen formation or allows its detection intraoperatively, thereby avoiding potential mortality. In the technique described by Qui et al., submucosal anastomosis occurred in one patient, requiring the re-canalization of the mucosa to restore luminal integrity [47].

In techniques that use OrVil or standard circular staplers for anastomosis, a median mini-laparotomy is necessary for inserting the stapler shaft. However, in our technique, we use only two EndoGIA linear staplers, eliminating the need for a median laparotomy incision. We retrieve the stomach and D1–D2 lymph nodes extracorporeally through an extended 12 mm left paramedian trocar incision. The incision size varies between 3 and 4.5 cm depending on the patient’s BMI. In the study by Wu et al., comparing traditional Roux-en-Y and pantaloon Roux-en-Y anastomoses, the average incision size was reported as 6.9 cm and 7.0 cm, respectively [43]. Compared to the existing literature, the incision size in our technique is significantly smaller. The smaller incision, positioned away from the midline, has also reduced our surgical site infection rate. Postoperative surgical site infection occurred in 2 of 21 patients (9.52%). Another advantage of the paramedian incision is its lower rate of incisional hernia compared to median incisions. Although the three-month follow-up period is relatively short, none of the patients exhibited signs of postoperative incisional hernias. The lower incidence of surgical site infection, faster wound healing, and absence of incisional hernia naturally contribute to a quicker return to daily life in the postoperative period without additional morbidity.

The success of a newly developed surgical technique cannot be measured solely by early or late postoperative clinicopathological outcomes. Factors such as ease of application, a short learning curve, procedure duration, and hospital and ICU stay also contribute to its success. We reviewed the literature on laparoscopic EJ anastomosis techniques, analyzing surgical duration, anastomosis time, and hospital stay. Wu et al. reported an average operative time of 212.5 min and a mean hospital stay of 12.8 days. In Guo et al.’s meta-analysis, the average operative time ranged from 199 to 309.14 min, the anastomosis time ranged from 45 to 58.11 min, and the average hospital stay was between 7.4 and 28.06 days [31,43]. In our technique, the average anastomosis time was 40.24 min, the total operative time was 180.48 min, and the mean hospital stay was 8.19 days. These results suggest that our technique is more practical, leading to a shorter operative time. However, a crucial point is the difference in operative time between the first and last cases in our series of 21 patients. While Wu et al. reported their findings based on 112 patients, and Guo’s meta-analysis included 29 to 139 patients per study, our first anastomosis took 50 min, whereas our last anastomosis took 30 min. The total operative time decreased from 210 min in our first case to 160 min in our last case. Although total surgical time is influenced by multiple factors beyond the anastomosis technique, these results highlight the short and simple learning curve of our method. In Antonakis’s study, the learning curve for laparoscopic distal gastrectomy was reported as 40–60 cases, while Jung et al. similarly suggested that laparoscopic total gastrectomy requires at least 50–60 cases to standardize [48,49].

In modern surgical practice, the cost of a procedure is significantly influenced by the surgical instruments used. While financial concerns should never take precedence over a patient’s life, economic realities differ across countries and societies. Regarding the costs of staplers used in gastrointestinal anastomoses, circular staplers are the most expensive. In our country, a circular stapler costs approximately USD 250, whereas an EndoGIA stapler costs USD 75. In LTG procedures, circular staplers and EndoGIA staplers are the most commonly used devices for anastomoses. Circular stapler-assisted anastomosis typically requires one circular stapler and two EndoGIA staplers on average [12]. The commonly preferred side-to-side EJ anastomosis technique described in the literature requires approximately three EndoGIA staplers [41]. Our anastomosis technique, however, uses only two EndoGIA staplers, resulting in an average cost of USD 150, whereas other anastomosis techniques range between USD 250 and 400. Thus, in addition to being easy to apply, our technique is also more cost-effective.

Due to its ease of application and short learning curve, the total laparoscopic intracorporeal esophagojejunostomy technique, known as “overlap”, and its modified version, the “modified overlap” technique, bear the closest resemblance to the SLEJ anastomosis technique we have developed. However, the overlap technique requires an additional linear stapler for stomach transection. Since the stomach is transected, the esophagus, characterized by a weaker wall structure, is used during traction. Moreover, as esophagotomy and jejunotomy are performed after transection and the common opening remains parallel to the anastomotic line, it must be hand-sutured. This necessity contributes to a higher risk of anastomotic complications, increased anastomosis time, prolonged total surgery duration, delayed anastomotic complications, and higher costs. The meta-analysis published by Guo et al. on the overlap anastomosis technique clearly demonstrates these disadvantages [31]. Compared to our SLEJ technique, the overlap technique exhibits significantly higher rates of operative time, anastomosis duration, intraoperative blood loss, hospitalization period, anastomotic leakage, stricture, and bleeding.

The only significant drawback of our anastomosis technique compared to other techniques is the higher incidence of pulmonary complications. We currently lack a definitive explanation for this observation. In our study, seven patients (33.3%) developed pulmonary complications. By comparison, pulmonary complication rates were 10% in Xing et al.’s study and 3.3–18.2% in Guo’s meta-analysis [31,32]. When comparing patient demographics (age, sex, comorbidities) with similar studies, we found no statistically significant differences. We hypothesize that the increased pulmonary complications in our technique may be attributed to using the stomach, which has a stronger wall structure, for traction. The stronger traction may induce temporary diaphragmatic dysfunction, impairing deep inspiration and expiration, leading to mucus accumulation and subsequent atelectasis. Therefore, we propose that preoperative instruction in respiratory exercises, combined with their consistent implementation both before and after surgery, may serve as an effective strategy to mitigate the increased incidence of postoperative pulmonary complications observed with this technique. By enhancing pulmonary function and promoting optimal lung expansion, such an approach could contribute to reducing the risk of atelectasis and improving overall respiratory outcomes in the postoperative period. In order to prevent these negativities, a guideline or a check list can be planned just like in patients who are planned for thoracotomy. The components of the guideline may include whether the patient is a smoker, the patient’s age, preoperative pulmonary capacity, a history of previous pulmonary infection, a history of previous thoracotomy operation, the presence of recent respiratory system disease, the patient’s body mass index, occupation (miner), and whether the patient has an active sports activity. With this check list, patients who are potentially prone to postoperative pulmonary complications can be identified and early precautions can be taken. Arterial blood tests, respiratory function tests, and cardiopulmonary exercise tests can be performed in suspicious patients. In this way, pulmonary risk maps of the patients are determined.

The major limitation of our study is that we did not perform a sufficient preoperative classification of the patients to be included in the study. When planning the reliability of our technique, we only considered the operation, anastomosis technique, and surgical complications. However, as we explained in the previous paragraph, we saw the most complications in the pulmonary system. We lacked a more detailed examination of patients with pulmonary system disease and preoperative rehabilitation of the respiratory system in these patients. This group of patients should be planned to be operated upon with other anastomosis techniques if necessary after sufficient examination.

## 5. Conclusions

The fundamental principle of laparoscopic surgery in cancer patients is to achieve the same oncological and survival outcomes as conventional surgery while yielding better early postoperative outcomes. We believe that we have developed an easily applicable, technically simpler, shorter-in-duration, easier-to-learn, and safer intracorporeal EJ anastomosis technique with a low rate of postoperative complications. Additionally, our technique does not require specialized instruments or materials; on the contrary, it is a cost-reducing method. This study presents a new method for laparoscopic EJ reconstruction and reports its early postoperative outcomes. In the second phase of our study, we plan to analyze overall survival, disease-free survival, and long-term anastomotic complications (such as stricture and recurrence) to provide a more comprehensive evaluation of the long-term outcomes of our technique.

## Figures and Tables

**Table 1:** Demographic and clinicopathological distribution of patients.

**Gender: n (%)**	
Male	**13 (61.9%)**
Female	**8 (38.1%)**
**Age, year, mean ± SD**	58.81 ± 7.89
**Height, centimeter, mean ± SD**	169.24 ± 8.92
**Weight, kilogram, mean ± SD**	75.39 ± 16.75
**Cigarette: n (%)**	
Absent	8 (38.1%)
Present	13 (61.9%)
**Comorbidity: n (%)**	
Absent	11 (52.4%)
Present	10 (47.6%)
**Comorbidity diseases: n (%)**	
Absent	11 (52.4%)
Cardiovascular disease	5 (23.8%)
Diabetus mellitus	3 (14.3%)
Cancer	2 (9.5%)
**Carcinoembryonicantigen, ng/mL, mean ± SD**	3.10 ± 1.65
**Albumin, g/dL, mean ± SD**	4.19 ± 0.27
**Hemoglobin, g/dL, mean ± SD**	11.78 ± 1.52
**Neoadjuvant treatment: n (%)**	
Absent	6 (28.6%)
Present	15 (71.4%)
**Tumor localication: n (%)**	
Siewert type 1	0 (0%)
Siewert type 2	9 (28.6%)
Siewert type 3	12 (71.4%)
**Tumor pathology: n (%)**	
Adenocancer classic variant	12 (57.2%)
Adenocancer ring-cell variant	5 (23.8%)
Neuroendocrine tumor	4 (19.0%)
**Tumor differentiation: n (%)**	
Grade 1	7 (33.3%)
Grade 2	6 (28.6%)
Grade 3	8 (38.1%)
**Lymph node dissection, number, mean ± SD**	24.81 ± 5.51
**Lymph node metastases, number, mean ± SD**	3.14 ± 4.24
**T stage: n (%)**	
T1	8 (38.1%)
T2	4 (19.0%)
T3	3 (14.3%)
T4	6 (28.6%)
**N stage: n (%)**	
n0	9 (42.9%)
n1	5 (23.8%)
n2	3 (14.3%)
n3	4 (19.0%)

**Table 2:** Surgical outcomes of patients.

**Operation time, minute, mean ± SD**	**180.48 ± 17.81**
**Anastomosis time, minute, mean ± SD**	40.24 ± 5.11
**Total stapler use, number, mean ± SD**	5.05 ± 0.21
**Anastomosis stapler use, number, mean ± SD**	2.05 ± 0.21
**Blood loss during anastomosis, milliliter, mean ± SD**	45.71 ± 27.03
**Maximum incision length, cm, mean ± SD**	3.28 ± 0.48
**Hospital stay, day, mean ± SD**	8.19 ± 2.13
**Anastomosis coast, dollar, mean ± SD**	158.57 ± 16.36
**Mortality: n(%)**	
Absent	21 (100%)
Present	0 (0%)
**Three-month weight loss, kg, mean ± SD**	3.24 ± 2.60

**Table 3:** Distribution of general and anastomosis technique complications.

**Complication: n(%)**	
Absent	**11 (52.4%)**
Present	**10 (47.6%)**
**General complications: n (%)**	
Surgical site infection	2 (20%)
Pulmonary complications	7 (70%)
**Anastomotic complications: n (%)**	
Anastomotic leakage	1 (10%)
Anastomotic bleeding	0 (0%)
Anastomotic stenosis	0 (0%)
**Clavien–Dindo classification, n (%)**	
Grade I	11 (52.4%)
Grade II	9 (42.9%)
Grade III	1 (4.7%)
Grade IV	0 (0%)
Grade V	0 (0%)

## Data Availability

The datasets used and/or analyzed during the current study are available primarily from the corresponding author on reasonable request (drsbmor@yahoo.com). If desired, databases could also be requested from other authors.

## Abbreviations

SLEJ, sutureless L-Shape EJ; EJ, esophagojejunostomy; TLTG, totally laparoscopic total gastrectomy; LDG, laparoscopic distal gastrectomy; LATG, laparoscopy-assisted total gastrectomy; LND, lymph node dissection; CME, complete mesogastric excision; GCL, gastrocolic ligament; AGB, arteria gastrica breves; LGA, left gastric artery; LHA, left hepatic artery; RGEA, right gastroepiploic artery; RGEV, right gastroepiploic vein; GDA, gastroduodenal artery; CHA, common hepatic artery; PV, portal vein; CT, celiac trunk; GEJ, gastroesophageal junction; DVT, deep vein thrombosis; PTE, pulmonary thromboembolism; MiNENs, mixed neuroendocrine non-neuroendocrine neoplasms.
